# P-1787. Tailor Swiftly: Lessons Learned from a Nationwide Implementation of an Antimicrobial Stewardship Program for Asymptomatic Bacteriuria

**DOI:** 10.1093/ofid/ofae631.1950

**Published:** 2025-01-29

**Authors:** Eva Amenta, Trenton M Haltom, John P Donnelly, Sophia Braund, Rogelio Hernandez, Andrew Chou, Tyler Brehm, Vanessa W Stevens, Barbara Trautner

**Affiliations:** Baylor College of Medicine, Houston, Texas; Baylor College of Medicine, Houston, Texas; University of Michigan, Ann Arbor, Michigan; Baylor College of Medicine, Houston, Texas; Baylor College of Medicine, Houston, Texas; Michael E. DeBakey Veterans Affairs Medical Center, Houston, Texas; Baylor College of Medicine, Houston, Texas; US Department of Veterans Affairs, Salt Lake City, Utah; Michael E. DeBakey Veterans Affairs Medical Center / Baylor College of Medicine, Houston, Texas

## Abstract

**Background:**

Overtreatment of asymptomatic bacteriuria (ASB) is a major cause of antibiotic overuse. We are facilitating an implementation of ASB Antimicrobial Stewardship (AS) intervention in 40 Veterans Affairs facilities, with 20 of these sites participating in a Virtual Learning Collaborative (VLC). The VLC meets twice monthly for webinars to hear from content experts and share stewardship strategies.

Figure 1.

**Figure:**
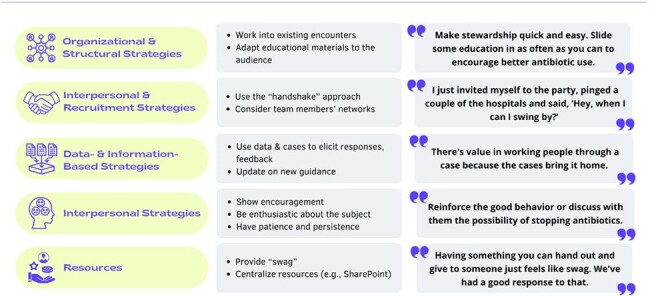

Thematic Categories of Stewardship Strategies for Implementation of Antimicrobial Stewardship Program

**Methods:**

Data come from descriptive field notes taken during each webinar. Notes focused on stewardship strategies shared by guest presenters or participating local site participants. Webinar sessions were recorded and transcribed using MS Teams. Transcripts were iteratively reviewed first by individual webinar, then across all webinars. Stewardship strategies were then organized thematically. We use exemplar quotes to illustrate and support thematic categories.

**Results:**

Across 19 webinars conducted between May 2023-February 2024, it became clear that each implementation site had different resources, team membership, and organizational structures. Thus, sites had to “tailor [the AS intervention] swiftly” for effective reach and implementation. Tailoring meant adapting the delivery of AS educational materials for timing, length, audience, and location of outreach. Sites used five strategies to implement the AS program: Organization and Structural Strategies, Interpersonal and Recruitment Strategies, Data- and Information-Based Strategies, Interpersonal Strategies, and Resources (**Figure 1**).

**Conclusion:**

VLC webinars encouraged sites to share tips and strategies about implementation of a nationwide AS program wherein rapid tailoring and focused strategies were effective. "Tailoring swiftly” acknowledges differing resources and organizational contexts to allow implementation sites to adapt AS materials to their needs.

**Disclosures:**

**Andrew Chou, MD, MSc**, Prolacta Bioscience: Advisor/Consultant **Barbara Trautner, MD, PhD**, Abbott Laboratories: Stocks/Bonds (Public Company)|AbbVie: Stocks/Bonds (Public Company)|Bristol Myers Squibb: Stocks/Bonds (Public Company)|Pfizer: Stocks/Bonds (Public Company)|Phiogen Pharma: Advisor/Consultant

